# Effects of composts obtained from hazelnut wastes on the cultivation of pepper (*Capsicum annuum*) seedlings

**DOI:** 10.1038/s41598-024-53638-4

**Published:** 2024-02-06

**Authors:** Faik Ceylan

**Affiliations:** Recycling of Agricultural Wastes to Industry Application and Research Center, Düzce University, Konuralp Campus, 81620 Düzce, Turkey

**Keywords:** Plant physiology, Environmental impact

## Abstract

Mixing animal waste and agricultural waste in certain proportions forms agricultural compost through appropriate air, time, and water supply. One of their use areas is directly used as fertilizer, and the other one is used as a material that can partially or completely replace P (peat) in the PGM (plant growth media). In this study, the initial mixtures with an appropriate C/N ratio and moisture content, which were created by mixing cow manure, chicken manure, hazelnut husk, hazelnut pruning wastes, vegetable and fruit wastes, and dry leaves, were composted for 180 days. The physicochemical properties of the mature composts were determined. Their effects on the fruit yield (weight of fruits) and plant height of pepper seedlings were evaluated in pot and field experiments. N (nitrogen), P (phosphorus), Cu (copper), and Zn (zinc) content were the highest in C4 (2.59%, 1.12%, 83.11 mg/kg, and 605.3 mg/kg). K (potassium) and Mn (manganese) content in C3 (1.79% and 750.5 mg/kg) and Fe (iron) content in C1 (4025 mg/kg) were determined to be the highest. There was no phytotoxic effect of all composts on *Lepidium sativum* seeds. Except for pH and organic matter, C1 45%, C1 20%, C4 45%, C4 20%, and P 90% met the requirements for ideal media. The mean height of eight-week seedlings increased in media of C1 20%, C1 45%, C2 20%, C2 45%, and C4 20%, but decreased in media of C3 90%. In field studies, while the highest yield was determined in C3 as 1530 g/plant, the lowest yield was 765.5 g/plant in control. The highest mean height was observed in C3 at 76.33 cm; the lowest was 63.03 cm in control.

## Introduction

Application of undecomposed or immature animal wastes to the soil causes immobilization of plant nutrients and phytotoxicity due to insufficient organic matter biodegradation^1^. By composting, the vitality of pathogens and seeds is eliminated, and the transformation of the high amount of heterogeneous solid organic matter into more stable and mature humic matter is achieved as a result of the activities of microorganisms^2^. Composting is one of the most effective and economical methods used for the recycling of organic waste^3^. It is known that composting animal wastes is very important in terms of both the prevention of environmental pollution and the production of organic fertilizers, which are value-added products^4^. Composed organic wastes are known as inexpensive soil conditioners and could be used as a plant nutrient source^1^.

In the last few years, hazelnut wastes have been studied to derive value from the production of medicinal mushrooms, biopellet production, etc.^5,6^. Composting is another way to add value to them. Agricultural wastes (hazelnut husk, hazelnut pruning waste, rice husk, etc.) are used as a carbon source for initial mixtures during composting. To our knowledge, there is not any study about composting of hazelnut pruning waste. Hazelnut husk and pruning waste, which are lignocellulosic materials, contain 15.4% and 34.7% cellulose, 25.9% and 25.4% lignin, 5% and 3% ash, 54.04% and 42.8% C, 0.97% and 0.64% N, 55 and 66.9 C/N, respectively^7–9^. Approximately 1.7 million tons of hazelnut pruning waste, which is a huge problem for hazelnut producers, are produced annually in Türkiye. All of it is burned for the purpose of house heating or disposal^5^. Although hazelnut husk, which is collected during harvesting, is used as a soil conditioner by some farmers, most of it is burned for disposal in the field. These activities cause air and soil pollution^8^.

Organic fertilizers (vermicompost, compost, microbial fertilizers, biofertilizers, etc.) are controlled releasing fertilizers that increase the microbial biomass of soil. On the other hand, mineral fertilizers (nitrate-based fertilizers, phosphorus fertilizers, potassium fertilizers, etc.) easily dissolve, and their nutrients disperse in the soil^10^. Synthetic fertilizers are used to increase crop yields, but their intensive use causes soil degradation and acidification, thus deteriorating soil fertility and product quality^11^. For this reason, the use of organic fertilizers is extremely important in terms of reducing the use of synthetic fertilizers and also providing a sustainable ecosystem by reducing the degradation of soil and water resources^11^. It is stated that the nutrients needed by the plants are provided as a result of the interaction of the living things in balance in the soil. For this reason, one of the most important methods to provide soil food web is the treatment of composted manure^12^.

While regulations of composts have some differences in different countries, pathogens like *Salmonella*, *E. coli, and* *Camylobacter* are not allowed to be in composts, or at least ≤ 2500 CFU/g for *E. coli*. Germination index of weeds should be more than 60%^13^. Heavy metal averaged limits of biowaste composts are 1.4, 93, 143, 1.0, 47, 121, 416, and 23 mg/kg for Cd (cadmium), Cr (chromium), Cu (copper), Hg (mercury), Ni (nickel), Pb (lead), Zn (zinc), and As (arsenic) in European countries^14^.

The quality of the matured compost formed as a result of the composting process is affected by the C/N ratios and moisture content of the starting materials^15^. For this reason, animal wastes with low carbon, high nitrogen content (low C/N ratio), and high humidity and agricultural wastes with high carbon, low nitrogen content (high C/N ratio), and low moisture content are mixed in appropriate proportions and matured composts are obtained in a short period of two months or even one month. Otherwise, they would sometimes take years under normal conditions. The recycling of agricultural wastes into compost fertilizers as high-value-added products is one of the important approaches that aim to minimize the damage caused by these wastes disposed of by incineration^16^.

In addition to their direct use in agricultural areas, composts could partially substitute peat, which is an expensive and non-renewable source for plant growth media, in soilless production at greenhouses^17^.

This study aims to obtain composts by composting hazelnut husk, hazelnut pruning wastes, fresh cow manure, and fresh chicken manure. In pot and field studies, it was hypothesized that these composts affected as much as chemical fertilizers on yield (fruit weight) and height of pepper seedlings and also increased the MB (microbial biomass) of soils.

## Materials and methods

### Preparation of initial mixtures for composting

Fresh cow and chicken manures were obtained from animal farms in Düzce province and its districts or from traditional livestock producers. On the other hand, agricultural wastes (hazelnut pruning waste and hazelnut husk) were obtained from the agricultural fields of the same region. Fresh cow or chicken manures and hazelnut pruning waste or hazelnut husk were composted in 1mx1mx1m (length × width × height) compost units at Duzce University, Recycling of Agricultural Wastes to Industry Application and Research Center (DUTAGAM) for 180 days from November 2022 to May 2023. The ratios of the components in the composts were adjusted according to^18^, considering the C/N (total organic carbon/total nitrogen) value of each component in Table 1. Initial mixtures as C1 (compost from hazelnut pruning waste and cow manure), C2 (compost from hazelnut husk and cow manure), C3 (compost from hazelnut husk and chicken manure), and C4 (compost from pruning waste and chicken manure), including component ratios, are presented in Table 2.

**Table 1:** C/N ratios of compost components.

Compost component	Fresh cow manure	Fresh chicken manure	Hazelnut husk	Hazelnut pruning waste	Vegetable wastes	Dry leaves
C/N ratio	25/1^4^	9.7/1^3^	55/1^8^	66.9/1^9^	~ 25/1^18^	~ 50/1^18^

**Table 2:** Ratios of components in initial compost mixtures.

Compost components (% w/w)	C1	C2	C3	C4
Fresh cow manure	55.53	59.37	–	–
Fresh chicken manure	–	–	35.57	32.67
Hazelnut husk	–	18.39	35.54	–
Hazelnut pruning waste	18.85	–	–	34.70
Vegetable wastes	22.25	19.28	26.62	28.58
Dry leaves	3.37	2.96	2.27	4.05

### Monitoring the composting process

During the composting process, the temperatures of the upper, central, and lower parts of the compost mixtures were measured every day, and a temperature graph was created using their averages. In addition, the temperatures just above the compost mixtures were measured as ambient temperatures during this period. Measurements of pH, EC, and determination of moisture contents of compost mixtures were done according to^4^. The mixtures of the samples to be taken from 4 points in total, from 2 points on the upper and lower parts of the compost mixtures, were mixed with distilled water at a ratio of 1:10 (w/v), and pH and EC were measured. Samples from 4 points for the moisture content of the compost mixtures were mixed, as in pH measurement, and measured using the oven-dry method at 105 °C. pH and EC values and moisture content of composts were measured on days 0, 2, 5, 8, 12, 18, 39, 49, 59, 69, 79, 89, 99, 109, and 119 during the composting process. The piles were aerated approximately every 10 days. At the end of the active composting, all compost piles were maintained at the maturation stage for an additional 90 days.

### Physicochemical analysis of composts

The N (total nitrogen) contents of matured composts were measured by the Kjeldahl method, and their TOC (total organic carbon) contents were calculated by dividing the OM (total organic matter) by 1,72 as a conversion factor. The OM was determined after burning 40 mesh compost materials in a muffle furnace at 550 °C. Measurements of P (total phosphorus), K (total potassium), Cu (total copper), Fe (total iron), Mn (total manganese), and Zn (total zinc) were made using ICP-OES according to the standard procedure of ISO 22036:2024. Certipur (Merck, Germany) was used as a certified reference material for ICP-OES calibration. Before ICP-OES measurements, samples were burned with a mixture of nitric acid and hydrogen peroxide in a microwave oven. Total humic (HA) and fulvic acid (FA) amounts of matured composts were determined according to^19^. The humification rate (HR) was calculated according to^20^ as follows:

$${\text{HR}} = \left[ {\left( {{\text{HA}} + {\text{FA}}} \right)/{\text{TOC}}} \right]*{1}00$$

### Phytotoxicity tests and seed germination index

Aqueous extracts were prepared from matured composts with a 1/10 (v/v) ratio using distilled water and filtered through filter paper for seed germination index (SGI)^21^. Eight milliliters of each extract were dropped onto Whatman #2 filter paper in petri dishes. Distilled water of eight milliliters was used as a control. Fifteen *Lepidium sativum* (purchased from Istanbul Seed Growing) seeds were put on the petri dishes and incubated at 21 ± 2 °C in the dark. Germinated seeds were counted five days later. The seed germination index was calculated using the following formula:

$${\text{SGI}}:\left( {{\text{Number of seeds germinated in extract}}*{\text{mean root length in extract}}} \right)/\left( {{\text{Number of seeds germinated in control}}*{\text{mean root length in control}}} \right)$$

### Physicochemical analysis of plant growth media and pepper cultivation in pots

C1, C2, C3, and C4 composts were mixed with peat or used alone for the preparation of plant growth media (v/v). All media have 10% sand in a volume. They were prepared and labeled as C1 90% (90% C1 + 10% sand), C2 90% (90% C2 + 10% sand), C3 90% (90% C3 + 10% sand), C4 90% (90% C4 + 10% sand), C1 45% (45% C1 + 45% peat + 10% sand), C2 45% (45% C2 + 45% peat + 10% sand), C3 45% (45% C3 + 45% peat + 10% sand), C4 45% (45% C4 + 45% peat + 10% sand), C1 20% (20% C1 + 70% peat + 10% sand), C2 20% (20% C2 + 70% peat + 10% sand), C3 20% (20% C3 + 70% peat + 10% sand), C4 20% (20% C4 + 70% peat + 10% sand), P 90% (90% peat + 10% sand). P 90% was used as a control. pH, EC, BD (bulk density), TP (total porosity), WHC (water holding capacity), and AC (aeration capacity) were determined according to methods in^22^. Organic matters of media were measured, like the physicochemical analysis of composts in this study.

Four pots were filled with plant growth media for each mix, and one 10-day pepper (cv. Yalova Carliston 341, purchased from Istanbul Seed Growing) seedling was sown in each pot. The height of seedlings was measured at 2, 4, and 8 weeks after the start of cultivation. Cultivation was managed from May to September 2023 in open field conditions. Seedlings were watered three times a week.

### Investigation of the effects of compost fertilizers on seedling growth in field

Field studies were carried out according to a randomized block design with 6 characters (C1, C2, C3, C4, 15 + 15 + 15, control) with 4 replications in Düzce/Türkiye from May to September 2023 for 4 months. Approximately 70 pepper seedlings were sown in each block. The blocks were arranged 2.1 m × 10 m in size on an area of 21 m^2^, with a total application area of 504 m^2^ and a total study area of 800.1 m^2^. The matured compost fertilizers and 15 + 15 + 15 (compound fertilizer, N + P + K) were calculated as 90 t ha^−1^ and 225 kg ha^−1^, respectively, according to N, P, and K values of soil at Table 3. 189 kg of compost fertilizers and 0.472 kg of 15 + 15 + 15 were applied to the blocks just before sowing seedlings. Compost fertilizers were laid the whole block equally. 15 + 15 + 15 was added to the under of seedlings. Morphological observations of the seedlings were made, according to^23^. At the end of the cultivation, the mean heights and mean fruit yields (weight of fruits) of 20 seedlings at each application were calculated.

**Table 3:** Properties of the soil used in the field experiments.

Parameter	Value
pH	6.12
Organic matter (%)	4.47
Available P (mg/kg)	35.92
Available K (mg/kg)	261.46
Total N (%)	0.22
Available Fe (mg/kg)	123.82
Cu (mg/kg)	2.9
Zn (mg/kg)	2.03
Mn (mg/kg)	13.22

### Microbial biomass

Microbial (bacteria and fungi) biomass contents from each matured compost and control soil were determined using the Microbiometer (USA) kit. In addition, 10 days from the start of the cultivation, in the middle of the cultivation, and at the end of the cultivation, the microbial biomass contents of soil samples from all field applications were measured.

### Statistical analysis

Differences between the microbial biomass of samples from each application at different times, mean fruit yields (fruit weight), and mean height of the seedlings of each application were analyzed by one-way analysis of variance using the GraphPad Prism 6 statistical program. Differences between means were compared using Tukey’s multiple-comparison test with a significance level of *P* < 0.05.

### Plant guideline statement

Experimental research and field studies on plants (either cultivated or wild), including the collection of plant material, comply with relevant institutional, national, and international guidelines and legislation.

## Results and discussion

### Monitoring of physicochemical parameters of composts

**Figure 1:**
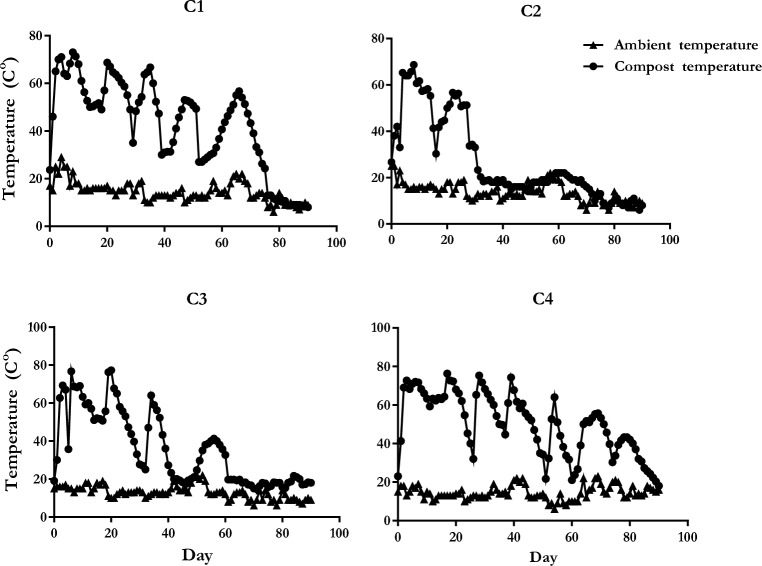
Evolution of temperature during composting process.

Measuring the temperature is one of the cheapest and easiest methods to observe the finalization of the bio-oxidative phase of composts^24^. The composting process includes mesophilic (10–42 °C), thermophilic (45–70 °C), mesophilic II (65–50 °C), and maturation (50–23 °C) stages^25,26^. The first three stages are known as the bio-oxidative phase. In this study, it was determined that the bio-oxidative phase took approximately 68, 27, 37, and 71 days in C1, C2, C3, and C4, respectively (Fig. 1). In the enclosed composting vessel^27^, reported that it took 41 days. The highest temperatures in C1, C2, C3, and C4 were measured as 73, 68, 69, and 75 °C, and the lowest temperatures were 8, 6, 14, and 18 °C, respectively. Toledo et al.^28^ showed that compost piles reached the top of thermophilic temperatures on two different days. In this study, C1 and C4 have four different days at the top of thermophilic temperatures. C2 and C3 had one and three days, respectively. It was considered that the thermophilic threshold and extension of the bio-oxidative phase in C1 and C4 compared to C2 and C3 could be related to the carbon source in the initial mixtures of them. Organic matter in hazelnut husk of C2 and C3 was stabilized earlier than hazelnut pruning wastes of C1 and C4.

**Figure 2:**
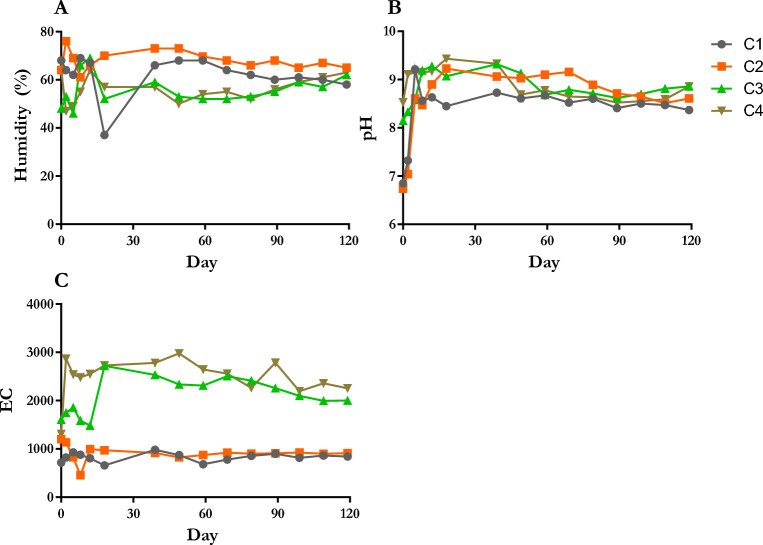
Measurements of (**A**) humidity, (**B**) pH, and (**C**) EC during composting process. EC: Electrical conductivity (µS/cm).

Humidity is one of the main factors in good composting because of the necessity for the survival of microorganisms in compost. It should be above 40% to not limit microbial activity^29^. The humidity of all composts in this study has remained between 37 and 76% (Fig. 2A). It was below 40% for 6 days in C1. In other composts, humidity was above 40% during the composting process. The pH of compost piles, which is an indicator for composting, is changed due to the degradation of organic matter and the mineralization of proteins and derivatives. In the whole process, this value must be maintained above 6^30^. pH has an important role in the activity of microorganisms, which need between 7 and 8.5 for optimum microbial activity^31^. The pH of the initial mixtures of C1 and C2 was under 7, but that of C3 and C4 was above 8 (Fig. 2B). At the beginning of the composting process, the pH of all composts increased quickly in 10–15 days and decreased slowly in the rest of the composting. At the end of the process, the pH of the composts ranged between 8.37 and 8.81. Another important indicator of the composting process is EC, which shows the soluble salt concentration. It must be under 4000 µS/cm so as not to be toxic to the crops^31^. C1, C2, C3, and C4 had stable EC after 15 days from the initial composting (Fig. 2C). While C1 and C2 had under 1000 µS/cm, C3 and C4 had above 2000 µS/cm but not more than 3000 µS/cm. It was determined that the pH and EC values of whole composts were within an adequate range for agricultural use.

### Physicochemical properties and microbial biomasses of composts

Humic substances (HS), including humic acid, fulvic acid, and humin, have great benefits in soil, like promoting nutrient uptake, and are correlated with the maturity of compost^32^. Zhu et al.^33^, and Huang et al.^34^ have reported that the HA + FA (humic and fulvic acid) contents of compost obtained from dairy and pig manure with pre-treatment of initial materials were 6.7% and 20%, respectively. In this study, we have determined that the HA + FA contents of composts were 20–26%. The highest value was 25.41 in C1. HR (humification ratio) is another indicator for the determination of composting efficiency and compost maturity^20^. Huang et al.^34^ showed that the HR of their composts was between 50 and 80. We have evaluated that this ratio of our composts was 61.03 as the highest value and 49.82 as the lowest value. These results were consistent with the previous studies and showed that all composts (C1, C2, C3, and C4) obtained in this study had composting efficiency from the frames of HS and HR (Table 4).

**Table 4:** Physicochemical properties and microbial biomasses of composts.

Parameter	Compost name			
	C1	C2	C3	C4
N (%)	1.56 ± 0.14	1.73 ± 0.12	2.07 ± 0.07	2.59 ± 0.23
TOC (%)	41.63 ± 1.84	41.41 ± 1.26	40.58 ± 1.29	37.40 ± 0.99
C/N	26.68 ± 1.35	23.93 ± 1.57	19.60 ± 1.27	14.44 ± 1.65
OM (%)	71.62 ± 3.18	71.23 ± 2.16	69.80 ± 2.22	64.33 ± 1.70
P (%)	0.33 ± 0.04	0.44 ± 0.09	0.99 ± 0.04	1.12 ± 0.11
K (%)	0.94 ± 0.13	1.74 ± 0.10	1.79 ± 0.15	1.54 ± 0.08
Cu (mg/kg)	16.26 ± 1.54	21.30 ± 1.47	64.45 ± 1.58	83.11 ± 2.07
Fe (mg/kg)	4025 ± 83.16	8879 ± 91.45	3889 ± 72.43	1775 ± 103.21
Mn (mg/kg)	289.2 ± 20.34	692.4 ± 34.45	750.5 ± 52.51	588.9 ± 24.76
Zn (mg/kg)	84.68 ± 12.41	141.3 ± 13.55	468.3 ± 10.58	605.3 ± 21.81
HA + FA (%)	25.41 ± 1.55	22.53 ± 1.23	20.22 ± 2.81	20.93 ± 1.55
HR	61.03 ± 4.42	54.40 ± 1.65	49.82 ± 6.39	55.96 ± 4.69
MB (ug C/g)	603 ± 25.55	551 ± 35.38	909 ± 96.86	1311 ± 82.27

Values after “±” indicate standart deviation.

Excessive heavy metal (Zn, Cu, etc.) concentrations are very toxic to soil and the cultivation of crops^35^. Therefore, there are some legislations for limiting these metal concentrations in composts. EU^14^ demonstrated that the average limits for the concentration of Cu and Zn in European countries were 143 and 416 mg/kg, respectively. While the Cu concentration of our entire compost was determined within the legal limits of the EU, that of Zn in C1 and C2 was seen to meet the requirements of EU legislation. It was revealed that Zn concentrations of C3 and C4 were higher than the limits of this legislation.

Composts obtained from organic wastes are a source of nutrients that can be utilized by plants. Regarding the plant nutrients, in our study, N and P content had the highest values in C4, K and Mn content in C3, and Fe content in C2. Not only this, but compost is a substrate for the microbiome, which could mineralize the organic matter^36^. It is stated that in microbiometer kit suppliers, while 1700 ug C/g and upper values indicate excellent microbial biomass for composts, 500 ug C/g and lower values show lower microbial biomass^37^. In our study, we determined C4 had the highest microbial biomass at 1311 ug C/g (Table 4). The microbial biomasses of all our composts were evaluated within this range.

During the composting process, the C/N ratio decreases because of the activity of microorganisms. The final C/N ratio generally depends on the components of initial mixtures, which indicates the maturity of composts^38^. Although this ratio is not enough to ensure, the final C/N ratio above 20 indicates the maturity of the compost. In this study, the C/N ratio was determined in C3 and C4 as 19.6 and 14.44, respectively (Table 4). It was determined that OM and TOC of C4 had the lowest values at 64.33 and 37.40, respectively (Table 4).

### Phytotoxicity tests and seed germination index

Seed germination index (SGI) is an indicator of the maturity and phytotoxicity of composts. More than 80% for the SGI value means that there is no phytotoxic effect on plants, and thus compost has maturity^39^. It was determined that all composts had no phytotoxic effect on the seed germination of *Lepidium sativum*. The SGI of C1 was revealed as the highest one at 134%, and the lowest one was that of C3 at 88% (Table 5).

**Table 5:** Seed germination index of composts.

Compost	SGI (%)
C1	134
C2	99.5
C3	88
C4	103.3

### Assessments on physicochemical parameters of plant growth media and pepper cultivation in pots

Necessities for ideal plant growth media are shown in Table 6, according to^40^. In this study, the highest values of pH and EC were measured in C2 90% as 8.22 and 1.03 mS/cm, respectively. In all plant growth media, it was observed that pH, EC, and bulk density gradually decreased with decreasing compost in the media. Whole plant growth media were not in the ideal range of pH and organic matter. That’s because the pH of all materials in the media was more than 7. Also, composts had no more than 70% organic matter, and plant growth media included inorganic matter such as sand. Total porosity (TP) was measured under 85% in C1 90%, C2 90%, C3 90%, and C4 90%. The highest value of water holding capacity (WHC) was determined in P 90% as 695.00 ml/L. The aeration capacities of plant growth media ranged between 19.27 and 43.93. Except for pH and organic matter, C1 45%, C1 20%, C4 45%, C4 20%, and P 90% met requirements for ideal media (Table 6).

**Table 6:** Physicochemical properties of plant growth media.

PGM	pH	EC (mS/cm)	BD (g/cm^3^)	TP (%)	WHC (ml/L)	AC (%)	OM (%)
Ideal^40^	5.3–6.5	≤ 0.5	< 0.4	> 85	600–1000	20–30	> 80
C1 90%	7.9 ± 0.09	0.74 ± 0.06	0.387 ± 9	83.35 ± 0.59	525.27 ± 32.41	30.83 ± 3.11	39.04 ± 1.59
C1 45%	7.31 ± 0.11	0.38 ± 0.05	0.334 ± 26	86.54 ± 1.67	634.07 ± 34.06	23.13 ± 1.91	39.36 ± 1.13
C1 20%	7.26 ± 0.08	0.27 ± 0.05	0.313 ± 20	87.05 ± 0.76	667.98 ± 42.56	20.25 ± 3.84	42.51 ± 1.98
C2 90%	8.22 ± 0.14	1.03 ± 0.12	0.402 ± 25	82.32 ± 1.03	383.93 ± 6.83	43.93 ± 0.88	35.76 ± 1.32
C2 45%	7.60 ± 0.11	0.55 ± 0.09	0.358 ± 31	86.80 ± 0.95	518.41 ± 10.51	34.96 ± 1.95	31.15 ± 2.18
C2 20%	7.37 ± 0.08	0.35 ± 0.03	0.301 ± 12	87.80 ± 0.75	532.43 ± 12.13	34.55 ± 0.59	38.85 ± 1.49
C3 90%	8.12 ± 0.09	0.80 ± 0.02	0.519 ± 19	80.41 ± 0.96	606.44 ± 31.50	19.77 ± 2.64	29.38 ± 1.75
C3 45%	7.88 ± 0.19	0.76 ± 0.02	0.341 ± 5	86.81 ± 0.57	561.15 ± 46.20	30.69 ± 4.27	35.21 ± 1.01
C3 20%	7.71 ± 0.03	0.36 ± 0.04	0.321 ± 35	87.64 ± 1.59	683.64 ± 4.14	19.27 ± 1.69	38.22 ± 1.27
C4 90%	7.99 ± 0.16	0.71 ± 0.02	0.432 ± 19	82.39 ± 1.19	534.24 ± 11.76	28.97 ± 1.40	34.53 ± 0.85
C4 45%	7.86 ± 0.08	0.52 ± 0.07	0.334 ± 6	87.01 ± 0.69	630.10 ± 45.67	24.00 ± 3.91	37.28 ± 1.11
C4 20%	7.06 ± 0.21	0.35 ± 0.09	0.358 ± 34	87.68 ± 1.19	629.02 ± 10.82	24.78 ± 1.63	29.93 ± 1.40
P 90%	8.89 ± 0.24	0.14 ± 0.02	0.250 ± 13	89.82 ± 0.78	695.00 ± 5.72	20.32 ± 1.29	44.48 ± 0.91

Values after “±” indicate standart deviation.

**Figure 3:**
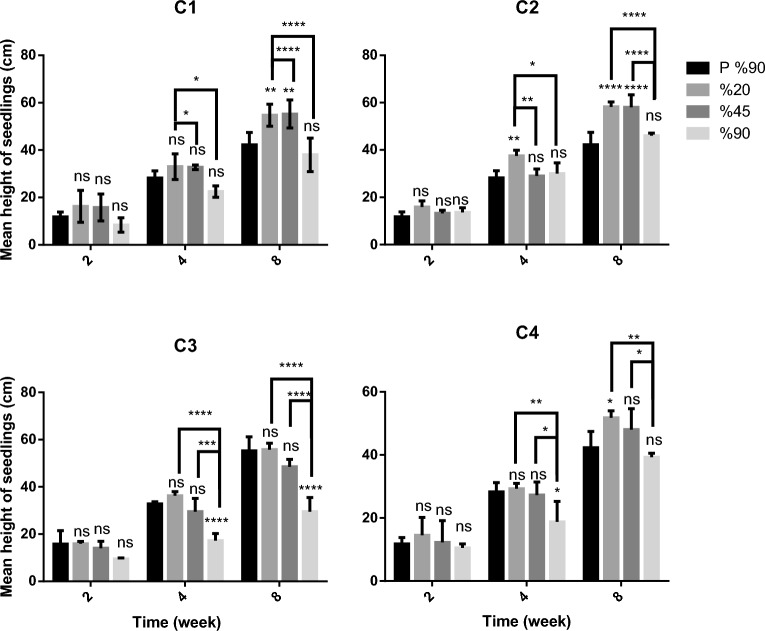
Mean height of pepper seedlings in different plant growth media. ****P < 0.0001, ***P < 0.001, **P < 0.01, *P < 0.05, ns: not significant.

Depending on their quality, adding compost materials to plant growth media could increase the plant's morphological characteristics, such as height^41^. For example^42^, showed that seedlings' height of *Gerbera jamesonii*, which was produced in media with 33% compost, increased compared to control media in the second week. In our study, it was revealed that there is no significant difference between the mean height of pepper seedlings in all growth media compared to P 90% in the second week (Fig. 3). It was reported that while tomato and cucumber transplants grown in homogenous composts died, the height of transplants grown in sphagnum moss substrate and their mixtures with composts was measured to be the tallest among other growth media at the fourth week^43^. Similarly, in our study, the mean height of pepper seedlings in media of C3 90%, C4 90% in the fourth week, and C3 90% in the eighth week decreased. In the fourth week, the mean height of pepper seedlings of C2 20% was significantly different compared to *P* 90% at the statistical level of *P* < 0.01. In the eighth week, the mean height of pepper seedlings in media C1 20%, C1 45%, and C2 20%, C2 45% were significantly different compared to *P* 90% at the statistical level of *P* < 0.01 and *P* < 0.0001, respectively. The seedling height of C4 20% was significantly different compared to *P* 90% at the statistical level of *P* < 0.05.

### Growth results of pepper plants at field studies

**Figure 4:**
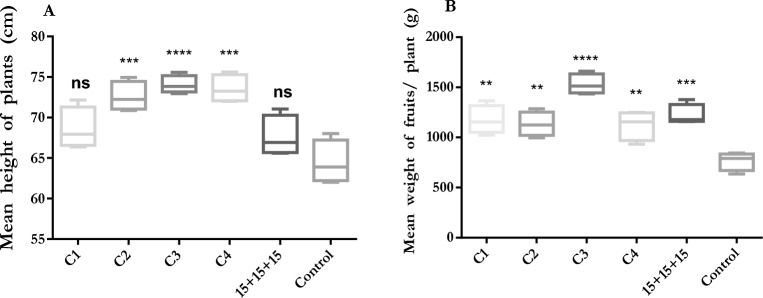
(**A**) Mean height of pepper plants (**B**) Mean weight of pepper fruits in field experiment. ****P < 0.0001, ***P < 0.001, **P < 0.01, ns: not significant.

There are some positive effects of composts, which are made from several organic waste materials, on improving crop yields. They are used generally in organic farming and the cultivation of vegetables by native people^43^. Khatun et al.^44^ found that composts obtained from sawdust and cattle manure mixtures, rather than only sawdust, increase the height of *Abelmoschus esculentus* (Okra) seedlings. It has been found that the application of vermicompost increases the height of pepper seedlings, which is 54 cm, compared to the control^45^. In our field study, it was revealed that the mean height of pepper plants was significantly different in C2 (*P* < 0.001), C3 (*P* < 0.0001), and C4 (*P* < 0.001) compared to control soil (Fig. 4A). However, there was no significant difference in C1 and 15 + 15 + 15 compared to the control. The highest mean height was 76.33 cm in C3.

Grain yields of wheat were determined to be 1452 kg/ha in control, doubled in both treatments of mineral fertilizer and traditional compost produced from cattle manure, and more than 4000 kg/ha in treatments of mineral fertilizer and traditional compost together^46^. In this study, there were significant differences in the mean weight of the fruits treated with C1 (*P* < 0.01), C2 (*P* < 0.01), C3 (*P* < 0.0001), C4 (*P* < 0.01), and 15 + 15 + 15 (*P* < 0.001) compared to the control soil (Fig. 4B). While the highest yield was determined in C4 at 1530 g/plant, the lowest yield was 765.5 g/plant in control. In previous studies, mean fruit yields were 800 g and 345 g in pots and 2227 g in field cultivations^45,47,48^. Our results show that there is consistency with other studies.

### Microbial biomass results in field studies

**Figure 5:**
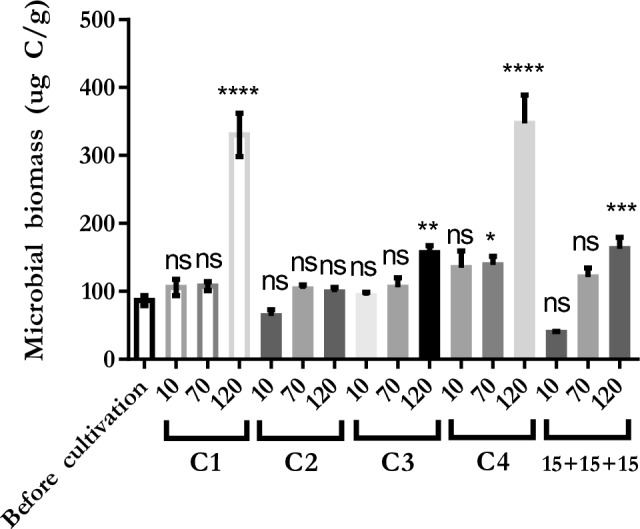
Microbial biomass of field soil before and 10, 70, and 120 days after cultivation. 10: 10 days after starting of cultivation, 70: 70 days after starting of cultivation, 120: 120 days after starting of cultivation, ****P < 0.0001, ***P < 0.001, **P < 0.01, *P < 0.05, ns: not significant.

It is stated that compost amendments could increase the microbial biomass of the soil^49^. Muhammad et al.^50^ have found that while the microbial biomass of soil treated with compost or humic substances was evaluated between 150 and 320 µg C/g, the control soil was 60 µg C/g. Nair and Ngouajio^51^ have also reported that soil with treated composts had higher microbial biomass than soil without any treatment. In this study, the microbial biomass of the soil was determined at 86 µg C/g before cultivation (Fig. 5). 120 days after cultivation, the microbial biomass of soil treated with C1, C3, C4, and 15 + 15 + 15 was evaluated as 330, 157, 347, and 162 µg C/g, respectively, and was determined to be significantly different (*P* < 0.0001, *P* < 0.01, *P* < 0.0001, and *P* < 0.001) compared to the microbial biomass of soil before cultivation (Fig. 5).

## Conclusions

All composts had no phytotoxic effect on *Lepidium sativum* seeds. Therefore, some heavy metal concentrations (Cu, Zn) of all composts that met EU legislation limits or were not excessive were consistent with phytotoxicity tests. On the other hand, there were some concerns (e.g., limited growth of pepper seedlings) about using these composts alone as the plant growth media. But they could be used as a partial substitute in growth media with peat. This substitution would decrease the costs of plant growth media for producers of pepper seedlings. All composts in this study, as well as compound fertilizer, increased the fruit yield of pepper during field studies. In future studies, seedlings of other species (tomato, aubergine, etc.) and trees from orchards can be cultivated with these composts in pot and field studies. Through the use of these compost fertilizers, excessive utilization of compound fertilizers would be limited. Thus, agricultural wastes (hazelnut pruning waste and hazelnut husk) could be recycled into value-added products by composting them.

## Data Availability

The datasets used and/or analysed during the current study available from the corresponding author on reasonable request.
